# Artificial Targets: a versatile cell-free platform to characterize CAR T cell function *in vitro*

**DOI:** 10.3389/fimmu.2024.1254162

**Published:** 2024-02-15

**Authors:** Xueting Wang, Nicholas J. A. Tokarew, Nadine Borgelt, Ramona Siemer, Cristiane Casonato Melo, Christian Langer, Ioannis Kasampalidis, Isabella E. Y. Ogusuku, Toni Cathomen, Isabel Gessner, Christian Dose, Jonathan A. Fauerbach, Anne Richter, César Evaristo

**Affiliations:** ^1^ Chemical Biology Department, R&D Reagents, Miltenyi Biotec B.V. & Co. KG, Bergisch Gladbach, Germany; ^2^ Institute for Transfusion Medicine and Gene Therapy, Medical Center – University of Freiburg, Freiburg, Germany; ^3^ Faculty of Biology, University of Freiburg, Freiburg, Germany; ^4^ Department of Biosciences and Medical Biology, Paris Lodron University of Salzburg (PLUS), Salzburg, Austria; ^5^ Center for Chronic Immunodeficiency (CCI), Faculty of Medicine, University of Freiburg, Freiburg, Germany

**Keywords:** CAR T cells, standardization, activation, functional assays, cell-free target, immunotherapy

## Abstract

Cancer immunotherapies using chimeric antigen receptor (CAR) T cells have tremendous potential and proven clinical efficacy against a number of malignancies. Research and development are emerging to deepen the knowledge of CAR T cell efficacy and extend the therapeutic potential of this novel therapy. To this end, functional characterization of CAR T cells plays a central role in consecutive phases across fundamental research and therapeutic development, with increasing needs for standardization. The functional characterization of CAR T cells is typically achieved by assessing critical effector functions, following co-culture with cell lines expressing the target antigen. However, the use of target cell lines poses several limitations, including alterations in cell fitness, metabolic state or genetic drift due to handling and culturing of the cells, which would increase variabilities and could lead to inconsistent results. Moreover, the use of target cell lines can be work and time intensive, and introduce significant background due to the allogenic responses of T cells. To overcome these limitations, we developed a synthetic bead-based platform (“Artificial Targets”) to characterize CAR T cell function *in vitro*. These synthetic microparticles could specifically induce CAR T cell activation, as measured by CD69 and CD137 (4-1BB) upregulation. In addition, engagement with Artificial Targets resulted in induction of multiple effector functions of CAR T cells mimicking the response triggered by target cell lines including cytotoxic activity, as assessed by exposure of CD107a (LAMP-1), expression and secretion of cytokines, as well as cell proliferation. Importantly, in contrast to target cells, stimulation with Artificial Targets showed limited unspecific CAR T cell proliferation. Finally, Artificial Targets demonstrated flexibility to engage multiple costimulatory molecules that can synergistically enhance the CAR T cell function and represented a powerful tool for modulating CAR T cell responses. Collectively, our results show that Artificial Targets can specifically activate CAR T cells for essential effector functions that could significantly advance standardization of functional assessment of CAR T cells, from early development to clinical applications.

## 1 Introduction

Chimeric antigen receptor (CAR) T cell immunotherapy involves infusion of genetically engineered T cells expressing CARs targeting specific disease-associated antigens (1). So far, CAR T cell therapy has demonstrated remarkable efficacy in the treatment of various hematological cancers (2–7) and represents a rapidly evolving field for treating other cancer types (8) as well as non-oncologic diseases (9, 10).

Eradication of cancer cells is associated with essential effector functions exerted by CAR T cells upon activation. These include T cell proliferation and expansion (11), cytotoxicity through degranulation of lytic granules or upregulation of ligands for death receptors to induce apoptosis of cancer cells (12), along with secretion of pro-inflammatory cytokines to recruit other immune cells and inhibit cancer cell growth (13, 14). Thus, functional characterization of CAR T cells is critical across all stages of cell therapy development.

Cell lines expressing the target antigen for CARs are conventionally used to activate and assess the functional capacities of CAR T cells *in vitro*. Yet, the handling and culturing of target cell lines might cause variations in metabolic cell states and expression of both target antigen and co-stimulatory ligands (CD80, CD86, 4-1BBL etc.) (15, 16) or inhibitory molecules (PD-L1, PD-L2, IDO etc.) (17–19). Together, these alterations could lead to different activation profiles of CAR T cells and create hurdles in comparing independent experiments. In addition, the mismatched expression of major histocompatibility complex (MHC) or human leukocyte antigens (HLAs) proteins between target cells and heterogenous CAR T cells may induce an allogenic response by the endogenous TCR, resulting in non-CAR-mediated T cell activation (20). To overcome these limitations and accommodate the needs for standardized *in vitro* functional assays, a synthetic system able to specifically activate CAR T cells via the CAR molecule and trigger important effector functions is desired. Such a synthetic system could provide numerous benefits including: increasing experimental control and reproducibility, reducing complexity in regard to handling of target cells, and preventing alloreactivity or limiting background signaling.

Thus far, limited cell-free platforms have been developed to specifically activate CAR T cells. The common point for the successful use of cell-free platforms in biological applications is the specificity of the platform in targeting the correct receptor(s) (21). A micropatterned antigen presenting surface with anti-idiotype antibodies and recombinant proteins of adhesion molecules has been reported to induce early activation signals and immunological synapse formation of CAR T cells (22). In addition, recombinant target proteins immobilized on surface plates (23) or on magnetic beads (24) could trigger antigen-dependent CAR T cell degranulation and cytokine secretion. These findings provided insights on the design of acellular target for CAR T cells. However, information about the response magnitude as compared to cellular stimulation remains to be elucidated. The signaling domain of a conventional second-generation CAR consists of a CD3 subunit and a costimulatory domain. Based on the CAR design, we hypothesized that crosslinking of CARs and thereby inducing CD3 and costimulatory signals could lead to sufficient CAR T cell response. Yet the prototype of Artificial Targets engaging only CAR molecules resulted in suboptimal CAR T cell activation that was lower than stimulation with target cell lines. Accumulating knowledge about CAR signaling (25–27) and emerging evidence suggest the requirement of endogenous costimulation for optimal CAR T cell activation (28, 29). Here, we developed Artificial Targets as a cell-free platform that mimicked target cell lines and activated CAR T cells in an antigen-dependent manner. This is achieved by simultaneously engaging CARs (via idiotype antibodies) and a costimulatory receptor, such as CD28, through the antibodies loaded on Artificial Targets. In addition, this platform is flexible as it allows changes of the target specificity and density by loading of different types and amounts of antibodies, which enables fine-tuning of CAR T cell responses in a standardized and controlled way. We demonstrate that Artificial Targets represent an effective platform for functional characterization of CAR T cells including phenotypic changes, degranulation, cytokine secretion and proliferation.

## 2 Materials and methods

### 2.1 Cell lines

JeKo-1 cell line was purchased from ATCC and transduced with lentiviral vector encoding firefly luciferase and eGFP under mouse phosphoglycerate kinase 1 promoter (PGK promoter), referring to the JeKo-1 WT cells used in this study. JeKo-1 CD19KO cell line was generated from the JeKo-1 WT cells with CRISPR Cas9 technology (specific guide: CCCCCCATGGAAGTCAGGCCCG) followed by single cell cloning with limiting dilution. JeKo-1 cell lines were cultured in RPMI 1640 medium supplemented with 10% FBS (EXIMUS) and 2 mM L-Glutamine (Gibco, Thermo Fisher).

### 2.2 Construction of CARs

Lentiviral CD19 CAR constructs (**Supplementary Figure 1A**) were synthesized under a human EF1-a promoter based on previously described design (30). CARs consist of a scFv derived from murine anti-CD19 antibody (clone: FMC63), a hinge region of CD8α, a transmembrane domain of TNFRSF19, an intracellular domain of either human CD28 or human 4-1BB, followed by the intracellular domain of CD3ζ.

### 2.3 Generation of CAR T cells

Anonymous human healthy donor buffy coats were purchased from Blutspendezentrale Dortmund or DRK Hagen. PBMCs were isolated using density gradient centrifugation with Pancoll human (PAN Biotech). T cells were then isolated using the Pan T Cell Isolation Kit (Miltenyi Biotec). To generate CAR T cells, bulk human T cells were activated on day 0 using T cell TransAct, human (Miltenyi Biotec) with 1:100 dilution in TexMACS medium (Miltenyi Biotec) supplemented with 10 ng/ml IL-7 and IL-15 (Miltenyi Biotec) (TexMACS complete medium). Cells were transduced with CAR lentivirus on day 1 at a multiplicity of infection (MOI) of 5. Meanwhile, untransduced (UTD) cells were cultured and expanded in the same way. On day 3, cells were washed with fresh TexMACS complete medium and resuspended in double volume of TexMACS complete medium for expansion. On day 6, samples were taken to assess transduction efficiency of CARs and the cell count, and fresh TexMACS complete medium was added. On day 10-12, CAR T cell isolation was performed with MACS isolation, for which 1 x 10^7^ cells were stained with 2 µl of anti-FMC63-PE antibody (Miltenyi Biotec, clone: REA1298) in CliniMACS PBS/EDTA Buffer (Miltenyi Biotec) with 0.5% BSA (Miltenyi Biotec) (PEB) buffer. Cells were washed 2x with PEB buffer and subsequently anti-PE MicroBeads UltraPure were added (Miltenyi Biotec) and applied onto MACS LS column for magnetic selection. The positive fraction containing CAR+ cells (enriched CAR T cells) were eluted in PEB buffer. The purity of isolated cells was examined by flow cytometry afterwards, and cells were centrifuged and cultured in TexMACS complete medium for at least 3 days prior to functional assays.

### 2.4 Generation and loading of Artificial Targets

Artificial Targets were generated by coupling of anti-Biotin antibody (Miltenyi Biotec, clone: REA746) to silica particles (Microparticles, 3.02 µm, SiO_2_-NH_2_-AR360) via Michael addition, as previously described (31–33). In short, antibodies were reduced using tris(2-carboxyethyl)phosphine hydrochloride (TCEP, Sigma-Aldrich) for one hour prior to the addition to SMCC activated beads. Conjugation was stopped after 2 h by addition of a 50 mM solution of β-mercaptoethanol (Calbiochem) followed by a 40 mM solution of N-ethylmaleimide (NEM, Sigma-Aldrich). Then antibody modified particles were collected via centrifugation (3000 x g, 5 min), washed with PBS/Pluronic (0.03%) and resuspended. Count of Artificial Targets was determined by MACSQuant Analyzer 10 (Miltenyi Biotec) and Artificial Targets were diluted to a concentration of 1.75 x 10^8^/ml with PBS. Loading of Artificial Targets was performed using a 1:1.2 ratio of anti-FMC63-Biotin (Miltenyi Biotec, clone: REA1297) antibody, and anti-CD28 Biotin (Miltenyi Biotec, clone: 15E8, functional grade) antibody, and/or anti-CD2 Biotin (Miltenyi Biotec, clone: LT2, functional grade) antibody in TexMACS GMP Medium (Miltenyi Biotec). Alternatively, biotinylated recombinat CD19-IgG1 was loaded on Artificial Targets (Miltenyi Biotec) together with anti-CD28 and anti-CD2.

### 2.5 Flow cytometry

Generally, flow cytometric acquisition for phenotypic characterization as well as flow-based functionality assays were performed on MACSQuant Analyzer 10. For determination of viable cell count, cells were stained with 1 μg/ml propidium iodide (PI) solution (Miltenyi Biotec) in culture medium prior to measurement. To assess the expression of activation markers, cells were incubated for 10 min at RT in the dark with antibodies diluted in PEB buffer and then washed twice with PEB buffer before measurement. For the staining of CD69 and CD137, 7-AAD staining solution was used together with the indicated antibody clones and fluorochromes (all from Miltenyi Biotec) as following: anti-CD3 VioBlue (clone: REA613), anti-CD69 PE (clone: REA824), anti-CD137 APC (clone: REA765), anti-CD8 VioGreen (clone: REA734), anti-CD4 APC-Vio770 (clone: REA623). In addition, fluorescence minus one (FMO) controls were included for analysis. All flow cytometry data were analyzed using FlowJo Software.

### 2.6 Degranulation assay

In each sample, 5 x 10^4^ effector cells (UTD or enriched CAR T cells) in 50 µl TexMACS GMP Medium were seeded onto a Falcon 96-well U-bottom plate. 25 µl CD107a staining mixture was added, consisting of 2 µl anti-CD107a APC (Miltenyi Biotec, clone: H4A3) and 200 nM Bafilomycin A1 (MedChemExpress) in TexMACS GMP Medium. Subsequently, 5 x 10^4^ Target cells or 1 x 10^6^ loaded Artificial Targets in 25 µl TexMACS GMP Medium were added to the effector cells. After a brief centrifugation at 80 x g for 1 min, the plate was incubated for 3 h in an incubator at 37°C with 5% CO_2_. The plate was centrifuged at 300 x g for 5 min and the culture supernatant was removed before 50 µl of the surface marker staining mixture (all from Miltenyi Biotec) was added, which contained anti-CD3 VioBlue (clone: REA613), anti-CD4 PE-Vio770 (clone: REA623), anti-CD8 APC-Vio770 (clone: REA734), 7-AAD staining solution, diluted in PEB buffer according to manufacturer’s instruction. The plate was briefly mixed and incubated in the dark for 10 min. Subsequently, 150 µl of PEB buffer was added for washing, the plate was centrifuged at 300 x g for 5 min and the supernatant was removed. The cell pellet was resuspended in 150 µl PEB buffer for acquisition on the MACSQuant Analyzer 10.

### 2.7 Confocal microscopy

A 96-well imaging plate (Miltenyi Biotec) was pre-coated with 100 µl of 100 µg/ml poly-D-lysine (Gibco, Thermo Fisher) for each well and incubated overnight at 37°C with 5% CO_2_. The plate was washed twice with 100 µl PBS (Gibco, Thermo Fisher) before adding the cells. CAR T cells were stained with CellTrace CFSE (Thermo Fisher) according to the manufacturer’s instructions. The stained CAR T cells were then mixed with UTD T cells from the same donor at a 1:1 ratio. Next, 1 x 10^6^ of the cell mixture was stained with 25 µl CD107a staining mixture described above (see “degranulation assay”). After 3 h of incubation, 50 µl surface marker staining mixture consisting of 1 µl anti-CD45 PE (Miltenyi Biotec, clone: REA747) in TexMACS GMP Medium was added to each sample. Cells were mixed and incubated at RT in the dark for 10 min. 150 µl of PEB buffer was added for washing (centrifugation at 300 x g for 5 min). After removing the supernatant, cells were resuspended in 100 µl TexMACS GMP Medium and transferred to poly-D-lysine coated imaging plate. The imaging plate was briefly centrifuged at 300 x g for 1 min prior to confocal microscopy on Zeiss LSM 710. Analysis was performed with ImageJ/Fiji.

### 2.8 MACSPlex for cytokine analysis

Cell-free supernatants from co-cultures of 2.5 x10^4^ effector cells with 1 x 10^4^ Target cells or 1.5 x 10^5^ loaded Artificial Targets were collected after 18 h (if not otherwise specified) of incubation and analyzed with the MACSPlex Cytotoxic T/NK Cell Kit, human (Miltenyi Biotec) or MACSPlex IFN-g Reagents Kit, human (Miltenyi Biotec) according to the manufacturer’s instructions. Levels of selected cytokines are shown. Automated analysis was performed with Express Modes on MACSQuantify Software.

### 2.9 Proliferation assay

Enriched CAR T or UTD T cells were labeled with 5 µM CellTrace Violet (Thermo Fisher) on day 0 following manufacturer’s instruction. 1 x 10^4^ stained cells were seeded as one sample on a 96-well U-bottom plate. T cells were cultured in different conditions: unstimulated, 1:500 diluted TransAct, 1 x 10^5^ Artificial Targets with different loadings, or with 1 x 10^4^ target cells. Triplicates of each sample and three identical plates were prepared, which were measured on day 5. Auto-labeling of PI was enabled for the acquisition on MACSQuant Analyzer 10.

### 2.10 Intracellular cytokine staining

In each sample, 5 x 10^4^ effector cells (UTD or enriched CAR T cells) in 100 µl TexMACS GMP Medium was firstly added onto a 96-well U-bottom plate. 5 x 10^4^ target cells or 1 x 10^6^ loaded Artificial Targets were resuspended in 100 µl TexMACS GMP Medium with 2 µg/ml Brefeldin-A (Sigma Aldrich) and subsequently added to the effector cells. After brief centrifugation at 80 x g for 1 min, the plate was incubated for 3 h in incubator at 37°C with 5% CO_2_. Viobility 488/520 Fixable Dye (Miltenyi Biotec) was prepared according to manufacturer’s instructions and diluted with PBS at 1:50. 50 µl of diluted Viobility Dye was added to all samples. After mixing, the plate was incubated at RT in the dark for 10 min. 50 µl of Inside Fix from the Inside Stain Kit (Miltenyi Biotec) was then added to all samples. The plate was incubated at RT in the dark for 10 min. 100 µl PEB buffer was added for washing (centrifugation at 300 x g for 5 min). After removing the supernatant, 200 µl Inside Perm from the Inside Stain Kit (Miltenyi Biotec) was added to wash the cells. Meanwhile, a staining mixture (all from Miltenyi Biotec) was prepared according to the manufacturer’s instructions, with 50 µl for one sample consisting of Inside Perm, anti-CD3 VioBlue (clone: REA613), anti-IFN-g APC-Vio770 (clone: 45-15), anti-TNF-a PE (clone: REA656), anti-IL-2 APC (clone: REA689), anti-CD4 PE-Vio770 (clone: REA623) and anti-CD8 PE-Vio615 (clone: REA734). The staining mixture was added to all samples and the plate was incubated in the dark at RT for 10 min. Cells were then washed by adding 150 µl Inside Perm (centrifugation at 300 x g for 5 min) and resuspended in 150 µl PEB buffer for measurement on MACSQuant Analyzer 10.

### 2.11 Whole transcriptome sequencing and analysis

1.5 x 10^6^ CAR T cells were cultured with either 1.5 x 10^6^ JeKo-1 WT cells or 7.5 x 10^5^ Artificial Targets loaded with anti-FMC63 and anti-CD28 antibodies for 2 h to avoid induction of secondary signaling pathways. Target cell depletion was performed with CD20 Microbeads, human (Miltenyi Biotec), and magnetically isolated sequentially with two LS columns (Miltenyi Biotec) according to manufacturer’s instruction. Cell count and purity were assessed by flow cytometry with MACSQuant Analyzer 10 (91-99.5% CD3^+^ GFP^-^). RNA was subsequently isolated from 1 x 10^6^ cells. RNA was extracted using RNeasy Mini Kit (QIAGEN). RNA yield was quantified using Qubit RNA HS Assay kit (Thermo Fischer Scientific) and quality was assessed with RNA 6000 Nano Chip (Agilent). Whole transcriptome libraries were generated using QIAseq Stranded mRNA kit (QIAGEN). The final libraries were quantified by Qubit dsDNA HS Assay kit (Thermo Fischer Scientific), Bioanalyzer High sensitivity DNA Assay (Agilent) and quantitative PCR with the NEB Next Library Quant Kit for Illumina (New England Biolabs). Sequencing was performed on Illumina MiSeq as QC run, and finally on Illumina NextSeq 550. Preprocessing of the data was conducted on CLC Genomics Workbench 23.0.2 (QIAGEN). RStudio was used for downstream analysis of the count matrix. The data was filtered for genes related to mitochondria or ribosome as well as genes with less than 50 counts across samples. Principal component analysis was performed on the 500 most variable genes. DESeq2 (34) (v.1.38.2) was used for differential gene expression analysis using the type of stimulation as model. Genes with FDR<0.01 and |Log2FC|>1 were considered differentially expressed. Gene set enrichment analysis was performed with ReactomePA using Log2FC for ranking (35) (v1.38.0).

### 2.12 Statistical methods

Data were analyzed with JMP 15 (SAS) and GraphPad Prism 9.1.2. The statistical tests used to calculate the P values are described in the respective figure legends. Unless otherwise stated, data were presented as mean ± SEM (Standard Error of the Mean). Significance was considered for P < 0.05 as the following: *P < 0.05, **P < 0.01, ***P < 0.001 and ****P < 0.0001. For experiments with multiple groups, multiple comparisons corrections were used as indicated in the figure captions.

## 3 Results

### 3.1 Artificial Target as a cell-free platform specifically activated CAR T cells *in vitro*

**Figure 1:**
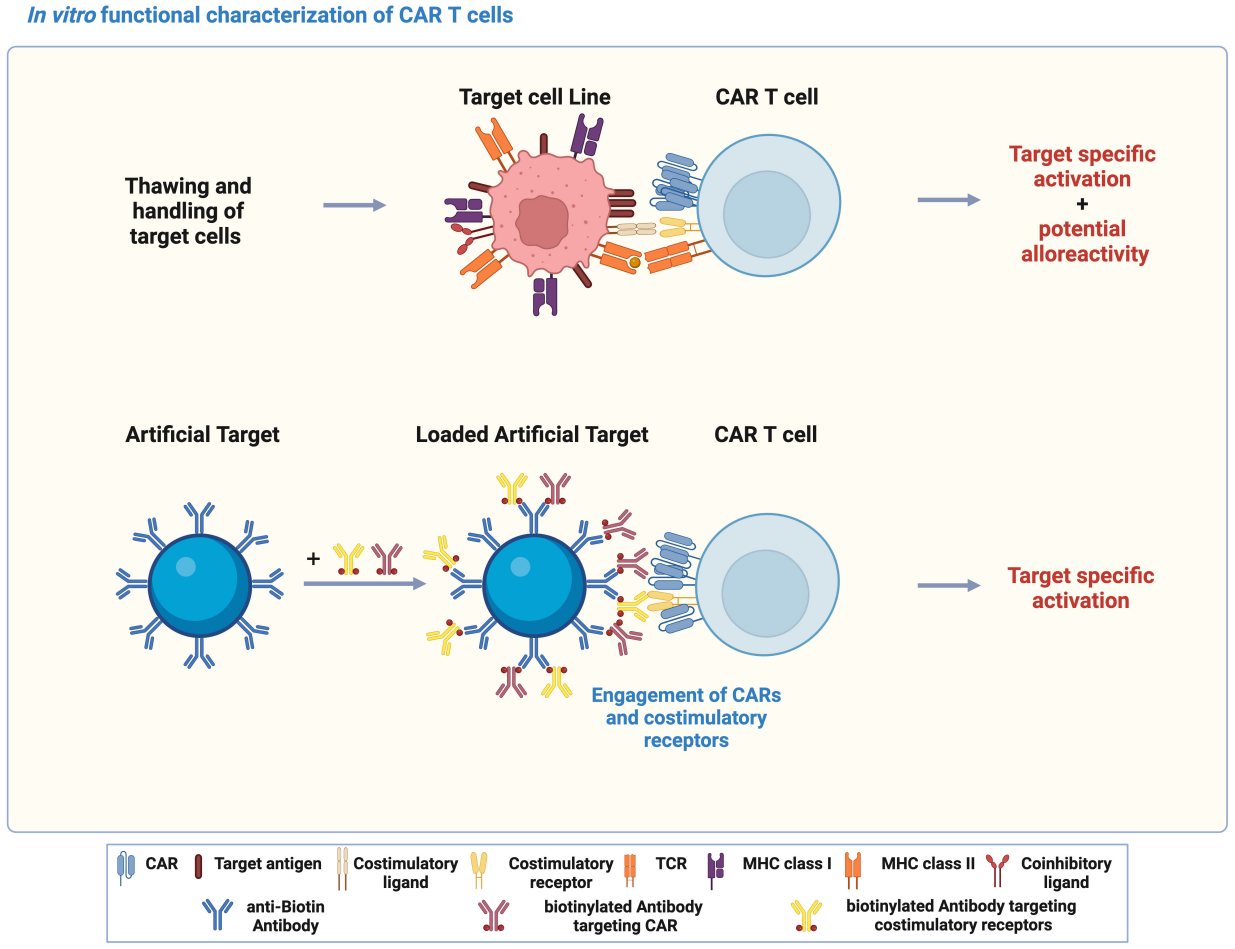
Artificial Targets as a cell-free alternative to activate CAR T cells *in vitro.* Schematic illustration of using target cell lines and Artificial Target for functional characterization of CAR T cells *in vitro*, and the principle of Artificial Targets. The illustration was created with BioRender.com.

*In vitro* functional characterization is critical for development of CAR T cells, which is typically achieved by co-culturing CAR T cells with target cell lines expressing the target antigen. However, variations in metabolic cell states and expression of both target antigen and co-stimulatory/inhibitory molecules might occur during handling and culturing of target cell lines. In addition, allogenic T cell response could also be triggered through the interaction between TCR and unmatched peptide-MHCs (**Figure 1**). In contrast to target cell lines, Artificial Target as a cell-free platform was designed to stimulate essential CAR signaling and activate CAR T cells specifically by defined antibody loading (**Figure 1**). In this study, Artificial Targets were prepared by covalently binding anti-biotin antibody to the surface of silica microparticles, and subsequent loading with biotinylated antibodies targeting CARs and costimulatory receptors. Engagement of CARs and costimulations induces CAR T cell activation, which can be assessed by different functional characterization.

**Figure 2:**
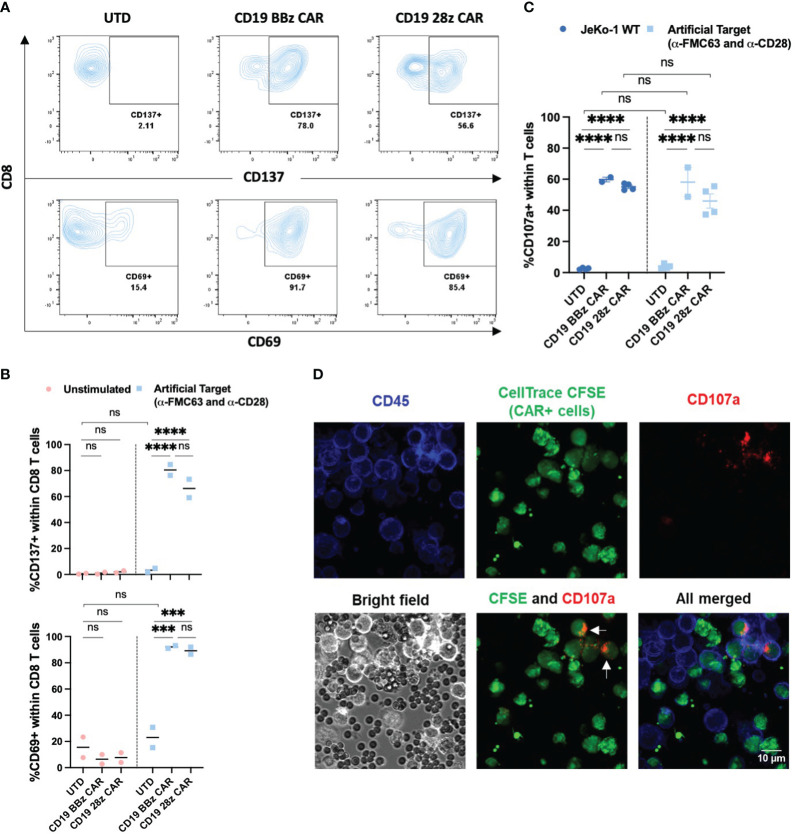
Expression of activation and degranulation markers upon stimulation. **(A)** Representative flow cytometric plots for CD137 and CD69 expression of CD8 T cells upon stimulation with Artificial Targets loaded with anti-FMC63 and anti-CD28 antibodies after 18 h incubation. **(B)** Summarized data of two donors for CD137 and CD69 expression. Data shown are two donors and their mean value. **(C)** CD107a expression of UTD (n=4), CD19 BBz CAR T cells (n=2) and CD19 28z CAR T cells (n=4) stimulated with JeKo-1 WT or Artificial Targets loaded with anti-FMC63 and anti-CD28 antibodies. Bars shown are mean ± SEM. **(D)** Representative images of confocal microscopy with 40x magnification. CAR+ T cells were stained with CellTrace CFSE and mixed with unstained UTD cells in a 1:1 ratio, and subsequently stained for CD45 after CD107a according to degranulation assay. Arrows pointed at CAR+ CD107a+ T cells. **(B, C)** Data were analyzed with two-way ANOVA and subsequently corrected by Tukey’s multiple comparisons test. ***P <0.001, ****P<0.0001; ns, not significant.

To determine whether Artificial Targets can specifically activate CAR T cells, we first examined the expression of activation markers CD137 and CD69 of untransduced (UTD) T cells and T cells engineered with two second-generation CARs, namely CD19 BBz CAR and CD19 28z CAR T cells (**Supplementary Figure 1A**), upon stimulation with Artificial Targets loaded with anti-FMC63 and anti-CD28 antibodies. As shown in **Figures 2A**, **B**, both CAR T cells showed significant upregulation of CD137 and CD69 in CD8 T cells when stimulated with Artificial Targets. In contrast, UTD cells demonstrated low expression of activation markers and the expression level remained comparable in unstimulated and stimulated conditions (**Figure 2B**), suggesting the benefit of Artificial Targets in preventing background signaling. Similar trends of CD137 and CD69 upregulation were observed in CD4 T cells (**Supplementary Figures 1B, C**). Our results show that Artificial Targets activated CAR T cells *in vitro* and induced CAR-specific upregulation of activation markers.

### 3.2 Artificial Targets induced CAR-specific degranulation

Several effector functions have been postulated to contribute to the effectiveness of CAR T cell in immunotherapy, such as cytotoxicity and production of cytokines. A major mechanism driving cytotoxicity relies on degranulation of pre-formed cytotoxic granules, containing the pore-forming protein perforin and serine proteases (granzymes), which are associated with lysosomal-associated membrane protein-1 (LAMP-1 or CD107) (36). Upon degranulation, perforin and granzymes are delivered to the target cell at the synapse region, and CD107a is exposed at the membrane of the effector cell (37). Hence, CD107a is associated with T cell-mediated cytotoxicity and represents a marker for degranulation (38). We have compared the CD107a expression upon cellular stimulation by CD19 expressing JeKo-1 mantle cell lymphoma cell line (JeKo-1 WT) with Artificial Targets loaded with anti-FMC63 and anti-CD28 antibodies. We observed a significant increase of CD107a expression in a CAR-dependent manner when stimulated with JeKo-1 WT and Artificial Targets within T cells (**Figure 2C**), as well as within the CD4 and CD8 T cell subsets (**Supplementary Figures 2A, B**, respectively). To note, the expression level of CD107a was comparable between cellular and artificial stimulation, and no significant difference was detected between the two different CAR constructs. CAR-specific degranulation was further confirmed by confocal microscopy to visualize the interaction between T cells and Artificial Targets, where CD107a expression was predominantly restricted to CAR+ T cells (pre-stained with CellTrace CFSE), as indicated by the arrows (**Figure 2D**). Together, these results demonstrate that Artificial Targets induce degranulation of CAR T cells.

### 3.3 Artificial Targets triggered cytokine expression by CAR T cells

**Figure 3:**
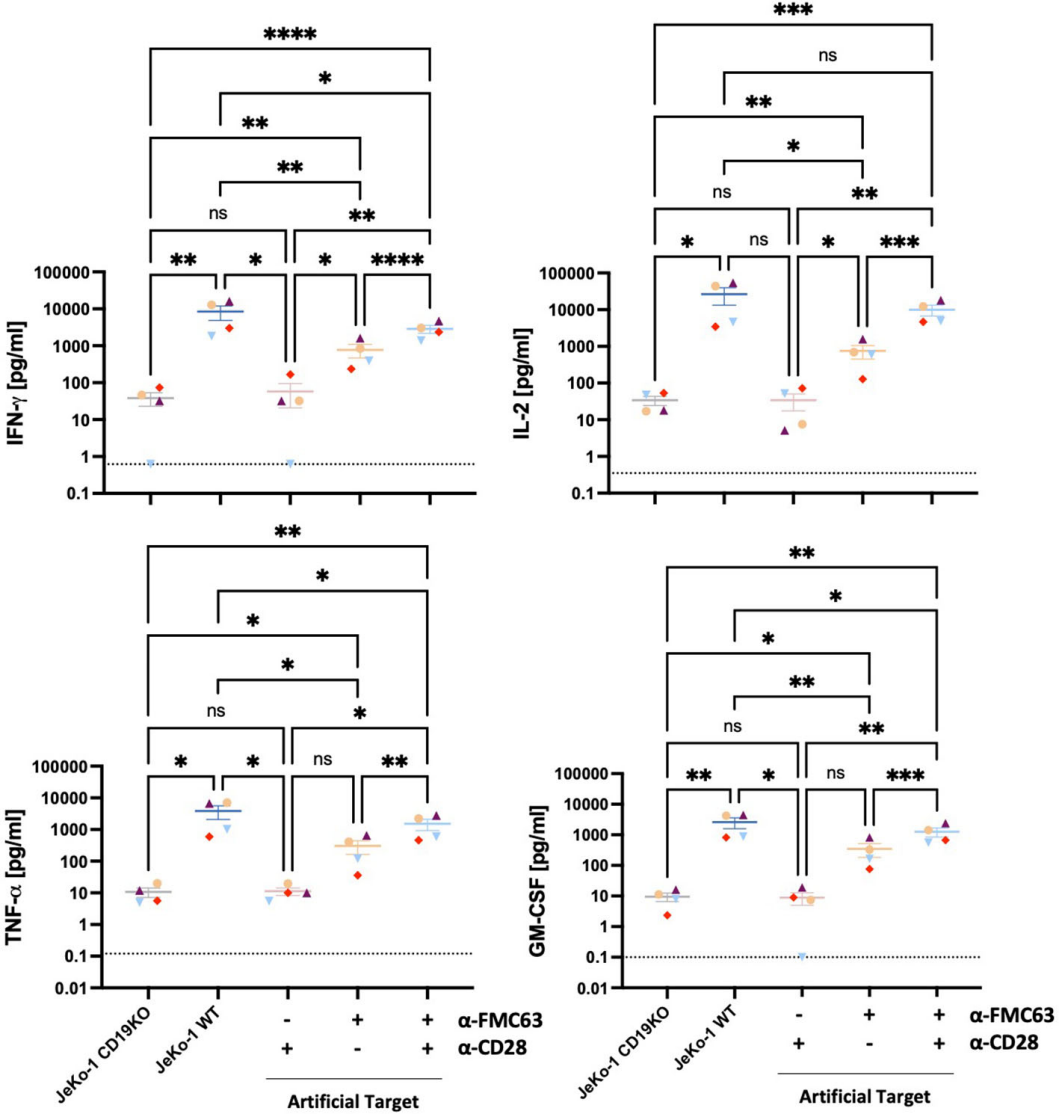
Artificial Targets triggered pro-inflammatory cytokine secretion of CAR T cells. Proinflammatory cytokine secretion (IFN-γ, IL-2, TNF-α, GM-CSF) of CD19 BBz CAR T cells (n=4) stimulated with JeKo-1 CD19KO, JeKo-1 WT or Artificial Targets with indicated loadings, respectively. Dashed lines indicate the detection limits of corresponding cytokines. Bars shown are mean ± SEM. Statistical analysis was performed by mixed-effects model with the Geisser-Greenhouse correction followed by Tukey’s multiple comparisons test. *P < 0.05, **P < 0.01, ***P < 0.001 and ****P < 0.0001; ns, not significant. Different colors/symbols indicate different donors.

We next assessed the potential of Artificial Targets to activate cytokine production. CD19 BBz CAR T cells were stimulated with Artificial Targets loaded respectively with anti-FMC63 and anti-CD28, only anti-FMC63 or only anti-CD28 antibodies. In addition, JeKo-1 WT cells and antigen-deficient JeKo-1 CD19KO cells were included as controls for cellular stimulations. While stimulation with JeKo-1 CD19KO and Artificial Targets loaded with anti-CD28 antibody resulted in limited CAR T cell response, CAR T cells treated with antigen-expressing JeKo-1 WT cells, Artificial Targets loaded with anti-FMC63 and anti-CD28 demonstrated CAR-specific secretion of IFN-γ, IL-2, TNF-α and GM-CSF (**Figure 3**). Moreover, Artificial Targets loaded with only anti-FMC63 antibodies triggered reduced CAR T cell response compared to JeKo-1 WT and Artificial Targets loaded with anti-FMC63 and anti-CD28. This is in line with the notion that endogenous costimulatory receptors along with CARs play an important role in CAR T cell activation (28, 29). To note, Artificial Targets loaded with anti-FMC63 and anti-CD28 antibodies were sufficient to trigger comparable IL-2 production as JeKo-1 WT cells and slightly lower level of IFN-γ, TNF-α and GM-CSF. Similar cytokine expression patterns were observed with intracellular cytokine staining (**Supplementary Figure 3**). Our data shows that Artificial Targets loaded with antibodies targeting CARs and costimulatory receptors can stimulate pro-inflammatory cytokine production of CAR T cells.

Next, we investigated whether Artificial Targets could fine-tune the CAR T cell response by adjusting the costimulations. CD2 as one costimulatory receptor binding to CD58 has been reported to positively modulate CAR T cell function *in vitro* and *in vivo* (28, 29). Therefore, we hypothesized that engaging CD2 could provide alternative or additional costimulation to CAR T cells. To test the hypothesis, Artificial Targets were differentially loaded by combining anti-FMC63, anti-CD28 and anti-CD2 antibodies, and the effect on pro-inflammatory cytokine secretion was evaluated (**Supplementary Figures 4A–D**). Overall, the cytokine secretion upon Artificial Targets loaded with only the costimulatory-element-targeting (CD2, CD28 or CD2 and CD28) antibodies resembled that of JeKo-1 CD19KO cells, showing low CAR T cell responses, whereas JeKo-1 WT and Artificial Targets loaded with anti-FMC63 together with one or two antibodies targeting costimulatory elements induced significantly higher secretion of IFN-γ, IL-2, TNF-α and GM-CSF. Significant increase of cytokine secretion was observed upon stimulation with Artificial Targets loaded with anti-FMC63 and anti-CD2 as opposed to Artificial Targets with only anti-FMC63 antibody. Notably, as compared to when only one costimulatory element (CD28 or CD2) was engaged together with CARs, CAR T cell responses were remarkably enhanced when Artificial Targets were simultaneously loaded with anti-FMC63, anti-CD28 and anti-CD2 antibodies, which resembles the stimulation of JeKo-1 WT cells. Moreover, we have employed recombinant fusion protein of human CD19 extracellular domain and a specifically mutated human IgG1 Fc region and observed comparable IFN-γ secretion as by using anti-idiotype FMC63 antibodies (**Supplementary Figure 2C**). Given the delayed expression of cytotoxic effector molecules (39), secretion of Granzyme B and Perforin upon stimulation with Artificial Targets could be assessed by prolonged incubation of 40 h (**Supplementary Figures 4E, F**). Concentrations of IL-6, IL-10 and MCP-1 were too low for reliable quantification. These data demonstrate the flexibility of this platform in activating and modulating CAR T cell response, and we could confirm the costimulatory role of CD2 in enhancing CAR T cell response.

### 3.4 Artificial Targets induced CAR T cell proliferation in an antigen-specific manner

**Figure 4:**
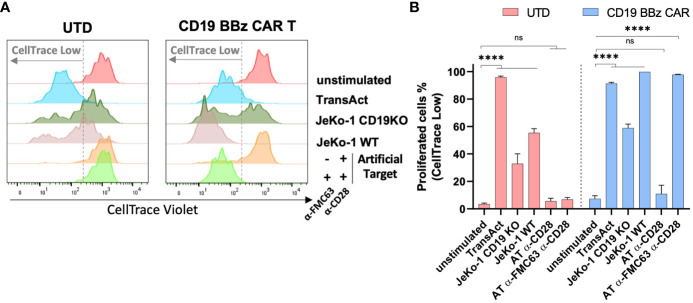
Artificial Targets induced CAR T cell proliferation in an antigen-specific manner. **(A)** Representative histogram overlays of CellTrace Violet labeled UTD and CD19 BBz CAR T cells after 5-day cultivation. **(B)** Representative frequencies of proliferated cells (indicated as CellTrace low fraction) of UTD and CD19 BBz cells in different conditions. Data shown are mean ± SD and analyzed with two-way ANOVA corrected by Dunnett’s multiple comparisons test. ****P < 0.0001; AT, Artificial Target; ns, not significant.

It has been shown that the proliferation and survival of CAR T cells strongly correlate with their antitumor efficacy *in vivo* (40). And we investigated whether Artificial Targets could be used to assess proliferative capacities of CAR T cells *in vitro.* To this end, CAR T cells were labeled with CellTrace dye and cell proliferation was monitored upon stimulation with JeKo-1 WT or JeKo-1 CD19KO cells, Artificial Targets with different loading or TransAct (polymeric nanomatrix conjugated to recombinant humanized CD3 and CD28 agonists that activate T cells). Furthermore, UTD cells were treated in the same way and served as a control. As shown in **Figure 4A**, divided cells could be clearly discriminated on day 5 based on the lower fluorescence intensity of CellTrace dye compared to unstimulated cells. Overall, we observed proliferation of CD19 BBz CAR T cells when stimulated with TransAct, JeKo-1 WT and Artificial Targets loaded with anti-FMC63 and anti-CD28 antibodies (**Figures 4A, B**). Interestingly, a sub-population of UTD cells proliferated when treated with both JeKo-1 WT and JeKo-1 CD19KO cells. In addition, a great proportion of CD19 BBz CAR T cells proliferated when treated with JeKo-1 CD19KO cells for 5 days, suggesting an antigen-independent response by alloreactive T cells that have been further enriched during the long-term culturing. In contrast, no remarkable proliferation of UTD cells was observed upon stimulation with Artificial Target regardless of the antibody loading. Together, these results show that Artificial Targets induce CAR T cell proliferation in an antigen-specific manner, and underscore the benefit of using Artificial Targets for functional characterization of CAR T cells due to the limited background activation.

### 3.5 Artificial Targets can simplify and reduce bias of transcriptomic analysis

**Figure 5:**
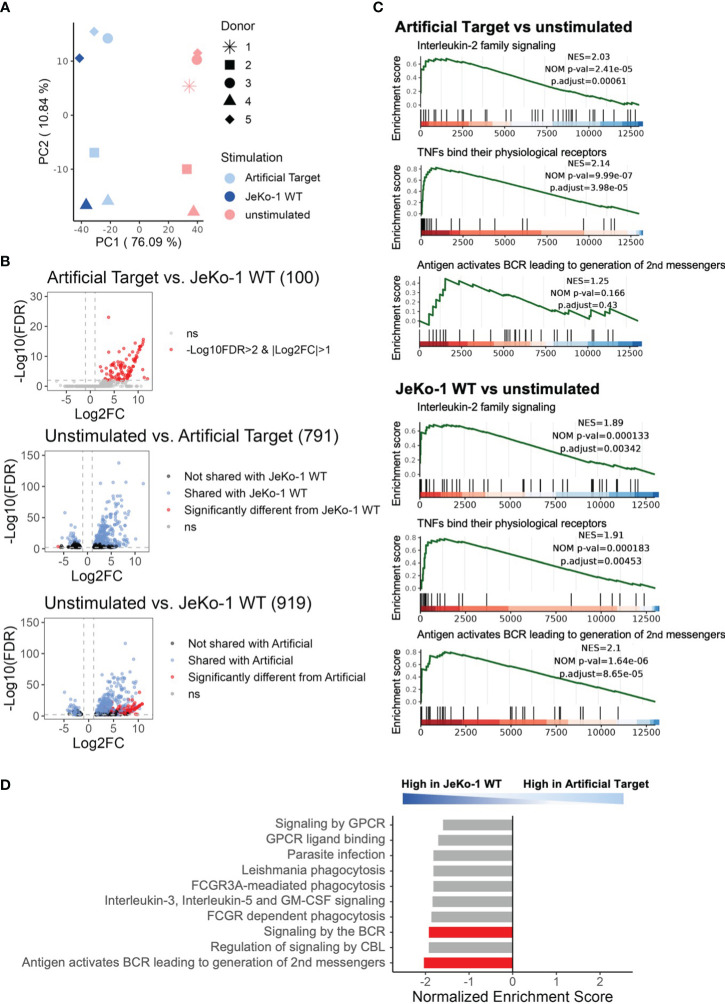
Using Artificial Targets for whole transcriptome sequencing. **(A)** Principal component analysis (PCA) plot of 500 most variable genes across samples. **(B)** Volcano plots of differentially expressed genes (DEGs) when comparing (upper) Artificial Targets vs. JeKo-1 WT stimulation, (middle) unstimulated vs. Artificial Targets, and (lower) unstimulated vs. JeKo-1 WT stimulation. The cutoffs of DEGs were set for |Log2FC|>1 and -Log10(FDR)>2. FDR, statistical approach used to adjust p-values for multiple testing; Log2FC, Log2 fold change. Numbers in brackets indicate the number of DEGs. **(C)** Gene Set Enrichment Analysis (GSEA) of DEGs of Artificial Targets (upper) and JeKo-1 WT (lower) stimulations for indicated pathways. NES, normalized enrichment score; NOM p-val, nominal p-value; p.adjust, adjusted (Benjamini-Hochberg) p-value. **(D)** Top 10 enriched pathways in the DEGs between JeKo-1 WT and Artificial Targets stimulations. Pathways enriched specifically for the stimulation were highlighted in red bars. **(C, D)** Gene set enrichment analysis was conducted with ReactomePA.

Finally, we performed whole transcriptome sequencing to compare the early transcriptomic signatures of CAR T cells between cellular and Artificial Target stimulations. To this end, unstimulated CAR T cells as well as CAR T cells stimulated with either Artificial Targets coated with anti-FMC63 and anti-CD28 antibodies or with JeKo-1 WT cells were cultivated for 2 h prior to RNA isolation. This early time point was selected to minimize noise resulting from secondary signaling events triggered by putative response of target cells to primary effector functions. Of note, significant loss of cell number was observed when performing target cell depletion in samples with JeKo-1 WT cells, resulting in limited samples for sequencing. In contrast, no additional purification steps are needed for samples stimulated with Artificial Targets. As expected, the principal component analysis (PCA) reveals similar expression between JeKo-1 and Artificial Targets stimulated cells compared to unstimulated samples (**Figure 5A**). The differential expression analysis confirmed that the differentially expressed genes (DEGs) upon Artificial Target stimulation were shared with JeKo-1 stimulation (**Figure 5B**) including upregulation of cytokines, such as IL-10, IL-31, IL-24, IL-3, IL-4, IL-2 and IL-13 (**Supplementary Figures 5A–C**). Despite the similarities, however, cells with cellular stimulation demonstrated unique transcriptomic signatures (**Figure 5B**, **Supplementary Figure 5A**). In addition, Gene set enrichment analysis (GSEA) revealed that “Interleukin-2 family signaling” and “TNFs bind their physiological receptors” were significantly enriched upon stimulation with Artificial Targets and JeKo-1 WT cells (**Figure 5C**). Notably, genes associated with “Signaling by the B Cell Receptor (BCR)” and “Antigen activates B Cell Receptor (BCR) leading to second messenger” were highly enriched in cells with JeKo-1 WT cells (**Figures 5C, D**). Moreover, a set of B cell specific genes, such as CD24, PAX5, MS4A1, was highly upregulated in samples stimulated with target cells, indicating the existence of B cells and creating difficulties in interpreting the data (**Supplementary Figure 5A**). Together, our data indicates that the early transcription profile induced by Artificial Targets is largely similar to that induced by cellular targets, and supports the use of Artificial Targets in RNA sequencing for simplified data preparation and clean readout.

## 4 Discussion and conclusion

The use of target cell lines in CAR T cell functional characterization presents several challenges in the development of robust and standardized assays. Target cell lines are subject to genetic drift during handling and passaging, leading to potential differences in results and creating hurdles in comparing experiments performed in multiple locations. As mitigation, large-scale cell banks can be generated, cryopreserved and maintained, e.g. in liquid nitrogen storage units. In addition, freezing, thawing and culture conditions may introduce variations in cell fitness and expression of key molecules that affect CAR T cell activities. A cell-free CAR T cell-activating platform has, thus, several advantages in reducing complexity and increasing reproducibility for functional characterization of CAR T cells. Acellular systems have been previously developed and proven useful to trigger antigen-specific degranulation and cytokine secretion as a measure for CAR T cell activation. Systems based on planar substrates, e.g., protein-functionalized 96-well plates or micropatterned surfaces, or using mobile bead-based platforms that resemble more closely cells have not yet explored the influence of costimulatory factors during CAR T cell activation (21–23). Other systems even tried to mimic the fluidity of cell membranes by covering a glass surface with a lipid bilayer (41) However, none of these platforms have demonstrated whether the induced CAR T cell activities were comparable to biologically relevant responses directed to B cell malignancies. While it is possible that a higher fluidity on acellular surface may facilitate interactions with cells, e.g., accessibility of specific surface proteins, it remains unclear whether this produces an effect, or whether a putative effect could be attributed to the fluidity or the overall difference in size, composition, and morphology of the employed systems. Our data showed that Artificial Targets demonstrate the capability to induce CAR T cell activation at a comparable level to the activation induced by target cells. We characterized this activation using multiple, orthogonal effector functions, including cytokine expression and secretion, degranulation and proliferation. Discrete transcriptional differences were identified in CAR T cells activated with Artificial Targets as compared to cellular targets. In addition to the possible contamination with small numbers of target cells, these differences could be attributed to the complex and highly variable molecular interactions with tumor cells. Individual patient tumor cells are phenotypically diverse, and may have different antigen and immunomodulatory molecule expression levels. Assessment of CAR T cell functionality can, thus, become highly dependent on the target cell line selected for analysis. Due to the acellular nature of Artificial Targets, CAR T cell activation can be reduced to the essential necessary molecular interactions, facilitating assay standardization.

As further development of CAR T cell therapy progresses, novel targets and CAR designs for treating different diseases emerge rapidly. Although we have focused on CD19 CARs in this work, Artificial Targets could be generated to activate different CARs with specific CAR-targeting molecules. For instance, natural ligands (CD19-Ig, recombinant fusion protein of CD19 and human IgG1 Fc domain) can be loaded on Artificial Targets and trigger equivalent CAR T cell activation (**Supplementary Figure 2C**), as well as anti-idiotype antibodies specific for other scFv of CARs (data not shown). In addition, Artificial Targets could be used for screening or measuring the role of the described endogenous costimulatory receptors, such as CD2 and CD28, or additional putative modulators, e.g. LFA-1, CD27 and 4-1BB (28, 29) in CAR T cell activities. Furthermore, the utilization of Artificial Targets represents a viable approach for studying the impact of inhibitory molecules on CAR T cells. Investigating not only the functional aspects but also the dysregulation of CAR T cells is a focal point of research in this field. For instance, these Artificial Targets could be loaded with purified proteins such as PD-L1 and Galectin-9 to respectively engage with PD-1 and TIM-3 receptors. Alternatively, they could be loaded with agonistic antibodies designed to target checkpoint receptors, like PD-1 (42). Additionally, antagonistic antibodies may also be employed to investigate the loss-of-function effects of particular molecules, such as the antagonistic antibody targeting CD28 known as lulizumab pegol (43). Moreover, Artificial Targets might provide standardized and controlled stimulus to other cell types developed for immune cell therapies, that include CAR natural killer (NK) cells (44), invariant NKT cells (45), or CAR γδ T cells (46).

So far, CAR T cell therapies have demonstrated unprecedented success in treating blood cancers. Yet, the translation of this novel approach to other types of disease remains challenging (47). It is believed that advanced knowledge in critical mechanisms regulating CAR T cell metabolic fitness, functionality and persistence would guide the widespread application of this novel therapy. CAR-mediated signaling and activation represents a subject of intense investigation in the field. Despite the similar effector functions shared between CAR T and TCR T cells, it has been shown that a CAR differs from a TCR in several ways across different stages of signaling transduction (26, 27), still with many open questions to be addressed. For instance, the signaling pathway involved in CAR T cell activation and the role of endogenous co-stimulation/-inhibition remain not fully understood. With the flexibility of Artificial Targets in engaging different or multiple molecules via targeted loading of respective agonistic or antagonistic entities, the use of Artificial Targets could help in dissecting the signaling pathways of defined molecular interactions without interference of unconcerned molecules.

Furthermore, CAR T cells treated with Artificial Targets instead of cellular targets provide the advantage of pure CAR T cell populations for subsequent downstream analysis and thereby increase the data quality. For example, when using target cells to assess the metabolic activities of CAR T cells upon activation, it’s challenging to interpret the metabolic profiles due to the active metabolism of target cells. In addition, Artificial Targets could facilitate the sample preparation for molecular analysis, such as RNA sequencing and western blot, as there is no need for removal of contaminating target cells.

In conclusion, Artificial Targets, as a cell-free platform, represents a powerful tool to characterize essential CAR T cell functionality. Thanks to the simple preparation procedure and flexibility in engaging defined molecules, we can envision a wide application of Artificial Targets in different research fields, from fundamental research to clinical development, with standardized readout.

## Data availability statement

The original contributions presented in the study are included in the article/**Supplementary Material**. Further inquiries can be directed to the corresponding author.

## Ethics statement

Ethical approval was not required for the studies on humans in concordance with the local legislation and institutional requirements because Buffy coats were provided by Klinikum Dortmund with written informed consent before sample collection and commercially available cell lines were used.

## Author contributions

XW: Conceptualization, Data curation, Formal analysis, Investigation, Methodology, Validation, Visualization, Writing – original draft, Writing – review & editing. NT: Conceptualization, Formal analysis, Investigation, Methodology, Visualization, Writing – review & editing. NB: Investigation, Writing – review & editing. RS: Investigation, Writing – review & editing. CM: Investigation, Writing – original draft, Writing – review & editing. CL: Methodology, Writing – original draft, Writing – review & editing. IK: Formal Analysis, Visualization, Writing – review & editing. IO: Methodology, Formal analysis, Visualization, Writing - review & editing. TC: Supervision, Writing – review & editing. IG: Methodology, Writing – original draft, Writing – review & editing. CD: Funding acquisition, Resources, Writing – review & editing. JAF: Methodology, Resources, Writing – original draft, Writing – review & editing. AR: Conceptualization, Funding acquisition, Methodology, Resources, Supervision, Writing – review & editing. CE: Conceptualization, Investigation, Methodology, Project administration, Resources, Supervision, Writing – original draft, Writing – review & editing.
