# Supplementary material for: Artificial Targets: a versatile cell-free platform to characterize CAR T cell function in vitro
Source: Front Immunol. 2024 Feb 15;15:1254162. doi: 10.3389/fimmu.2024.1254162 (PMC10906080; doi:10.3389/fimmu.2024.1254162)
Supplement: Supplementary file 1 [file DataSheet_1.docx]

Supplementary Material

**Artificial Targets: a versatile cell-free platform to characterize CAR T cell function *in vitro***

Xueting Wang, Nicholas J. A. Tokarew, Nadine Borgelt, Ramona Siemer, Cristiane Casonato Melo, Christian Langer, Ioannis Kasampalidis, Isabella E.Y. Ogusuku, Toni Cathomen, Isabel Gessner, Christian Dose, Jonathan Fauerbach, Anne Richter and César Evaristo^*^

*** Correspondence:** César Evaristo: cesare@miltenyi.com

# Supplementary Figures

##


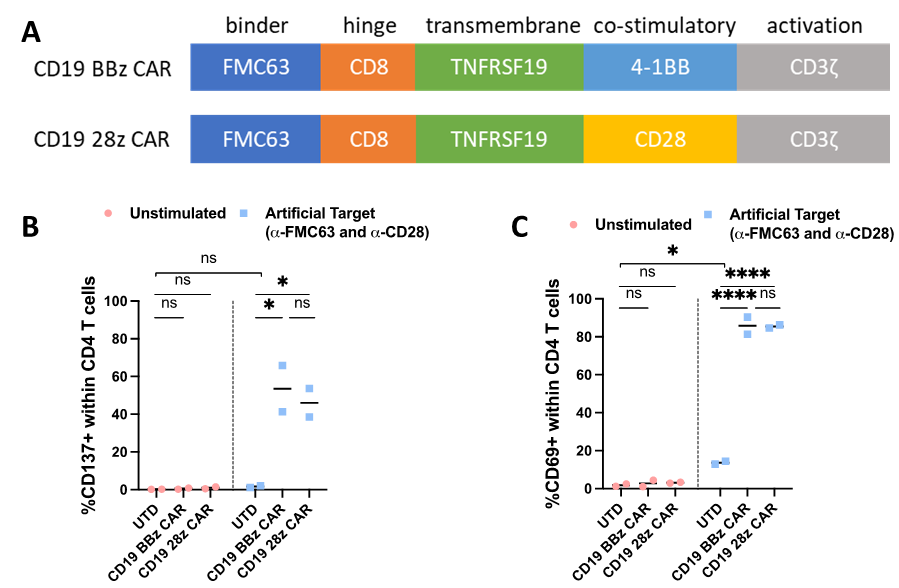


**Supplementary Figure 1:**

**(A)** Design of CAR constructs used in this study. **(B)(C)** Summary data of **(B)** CD137 and **(C)** CD69 expression in CD4 T cells (n=2). Bars shown are mean values from two donors and analyzed with two-way ANOVA and subsequent multiple comparisons, corrected by Tukey’s multiple comparisons test. * P < 0.05, **** P < 0.0001; ns, not significant.

**Supplementary Figure 2:**

Respective CD107a frequencies of **(A)** CD4 and **(B)** CD8 cell subsets from UTD (n=4), CD19 BBz CAR T cells (n=2) and CD19 28z CAR T cells (n=4) stimulated with JeKo-1 WT or Artificial Target loaded with anti-FMC63 and anti-CD28 antibodies. Bars shown are mean ± SEM, analyzed with two-way ANOVA and subsequent multiple comparisons, corrected by Tukey’s multiple comparisons test. **(C)** IFN- γ secretion of CAR T cell only, upon stimulation with CD19- or CD19+ JeKo-1 cells, or with Artificial Targets with indicated loadings (n=2). CD19-IgG1, recombinant fusion protein of human CD19 extracellular domains and a specifically mutated human IgG1 Fc region. Dashed line indicates the detection limit. Bars shown are mean ± SEM. Statistical analysis was performed by mixed-effects model followed by Tukey’s multiple comparisons test. **** P < 0.0001; ns, not significant.

##

**Supplementary Figure 3:**

Intracellular staining of IFN-γ **(A)(B)(C)**, IL-2 **(D)(E)(F)** and TNF-α **(G)(H)(I)**. Summary data of UTD (n=4), CD19 BBz CAR T cells (n=2) and CD19 28z CAR T cells (n=4) stimulated with JeKo-1 CD19KO (grey circle), JeKo-1 WT (dark blue square) or Artificial Target loaded with anti-CD28 (beige triangle) or with anti-FMC63 and anti-CD28 antibodies (light blue circle). Frequencies in T cells **(A)(D)(G)**, CD4 T cells **(B)(E)(H)**, or CD8 T cells **(C)(F)(I)** were presented, respectively. Bars shown are mean ± SEM and analyzed with two-way ANOVA and subsequent multiple comparisons, corrected by Dunnett’s multiple comparisons test. *P < 0.05, **P < 0.01, ***P < 0.001 and ****P < 0.0001; ns, not significant.

##

**Supplementary Figure 4:**

Secretion of **(A)** IFN-γ, **(B)** IL-2, **(C)** TNF-α and **(D)** GM-CSF by CD19 BBz CAR T cells (n=4) stimulated with JeKo-1 CD19KO, JeKo-1 WT or Artificial Targets with indicated loadings, respectively. Dashed lines indicate the detection limits. Bars shown are mean ± SEM. Statistical analysis was performed by mixed-effects model with the Geisser-Greenhouse correction followed by Tukey’s multiple comparisons test. Different colors/symbols indicate different donors. Secretion of cytotoxic effector molecules Granzyme B **(E)** and Perforin **(F)** by CAR T cell only, or CAR T cells stimulated with Artificial Targets bearing indicated loadings after prolonged co-culture (40 h). Dashed lines indicate the detection limits. Statistical analysis was performed by ordinary one-way ANOVA followed by Dunnett’s multiple comparisons test. *P < 0.05, **P < 0.01, ***P < 0.001 and ****P < 0.0001; ns, not significant.

**Supplementary Figure 5:**

**(A)** Heatmap of the top 35 down-regulated (upper panel) and up-regulated (lower panel) genes of JeKo-1 WT stimulated cells vs. unstimulated cells identified by differential expression analysis (DESeq) with cut-offs of FDR<0.01 and |Log2FC|>1. Data included unstimulated CD19 BBz CAR T cells (n=5), CAR T cells stimulated with Artificial Target (n=4) as well as CAR T cells isolated from co-culture with JeKo-1 WT cells (n=2). Gene expression was scaled and demonstrated by z-score. Donors, conditions and names of DEGs were color-coded as indicated in the legends. **(B)** Top 15 enriched pathways of upregulated DEGs of Artificial Target stimulation vs. unstimulated cells. **(C)** Top 15 enriched pathways of upregulated DEGs of JeKo-1 WT stimulation vs. unstimulated cells. **(B)(C)** Gene set enrichment analysis was conducted with ReactomePA with cut-off of adjusted (Benjamini-Hochberg) p-value < 0.05. Bars colored in blue indicate shared pathway between Artificial Target and JeKo-1 WT stimulations, whereas bars colored in red indicate specific pathways.
